# Type 1 diabetes differentially disrupts right and left ventricular daily rhythms

**DOI:** 10.1152/function.041.2026

**Published:** 2026-10-01

**Authors:** Sharanya S. Bettadapura, Samantha J. Torres, Marjie P. Schmitt, Brandon L. Roberts, Danielle R. Bruns

**Affiliations:** ^1^Department of Zoology & Physiology, 4416 University of Wyoming, Laramie, Wyoming, United States; ^2^Program in Neuroscience, University of Wyoming, Laramie, Wyoming, United States

**Keywords:** circadian rhythms, left ventricle, right ventricle, type I diabetes

## Abstract

Circadian rhythms are endogenous ∼24-h cycles that regulate cardiovascular physiology. Although circadian regulation of the left ventricle (LV) is well established, whether the right ventricle (RV) exhibits intrinsic rhythmicity is not known. Here, we provide the first evidence that the healthy RV exhibits robust rhythms in function and molecular gene expression. Cardiovascular disease is the leading cause of mortality in type 1 diabetes (T1D). T1D disrupts circadian rhythms, yet how T1D alters chamber-specific circadian control remains unclear. We investigated RV and LV function and gene expression across the 24-h light-dark cycle in male streptozotocin-induced T1D and control mice. Echocardiographic assessment of diabetes-induced remodeling at ZT0–4 and ZT12–16 [zeitgeber time (ZT)] revealed time-dependent functional impairment, including reversal of the normal diurnal heart rate pattern. T1D impaired day-night RV and LV systolic function, with loss of day-night difference in LV ejection fraction and impaired active period RV stroke volume. Cosinor analysis of RV and LV gene expression demonstrated preserved 24-h rhythmicity of core clock genes *Arntl1* and *Per2* in both ventricles with T1D, whereas the clock output genes *Nr1d1* and *Dbp* had dampened amplitude in the RV but amplified expression in the T1D LV. These findings demonstrate that T1D differentially disrupts circadian regulation of the RV and LV, with selective vulnerability of the clock-controlled output genes despite preservation of the core oscillator. Chamber-specific circadian remodeling may contribute to the elevated cardiovascular risk in T1D and has implications for the timing of diagnostic and therapeutic interventions.

**NEW & NOTEWORTHY** This study provides the first evidence that the healthy right ventricle (RV), like the left ventricle (LV), possesses an intrinsic circadian clock with robust 24-h rhythmicity. Using a mouse model of type 1 diabetes, we show that diabetes does not uniformly suppress cardiac clocks but instead differentially rewires RV and LV rhythms, dampening clock-output gene amplitude in the RV while amplifying it in the LV. Together, we reveal chamber-specific circadian vulnerability with implications for diabetic cardiomyopathy and chronotherapy.

## Graphical abstract

**Figure:**
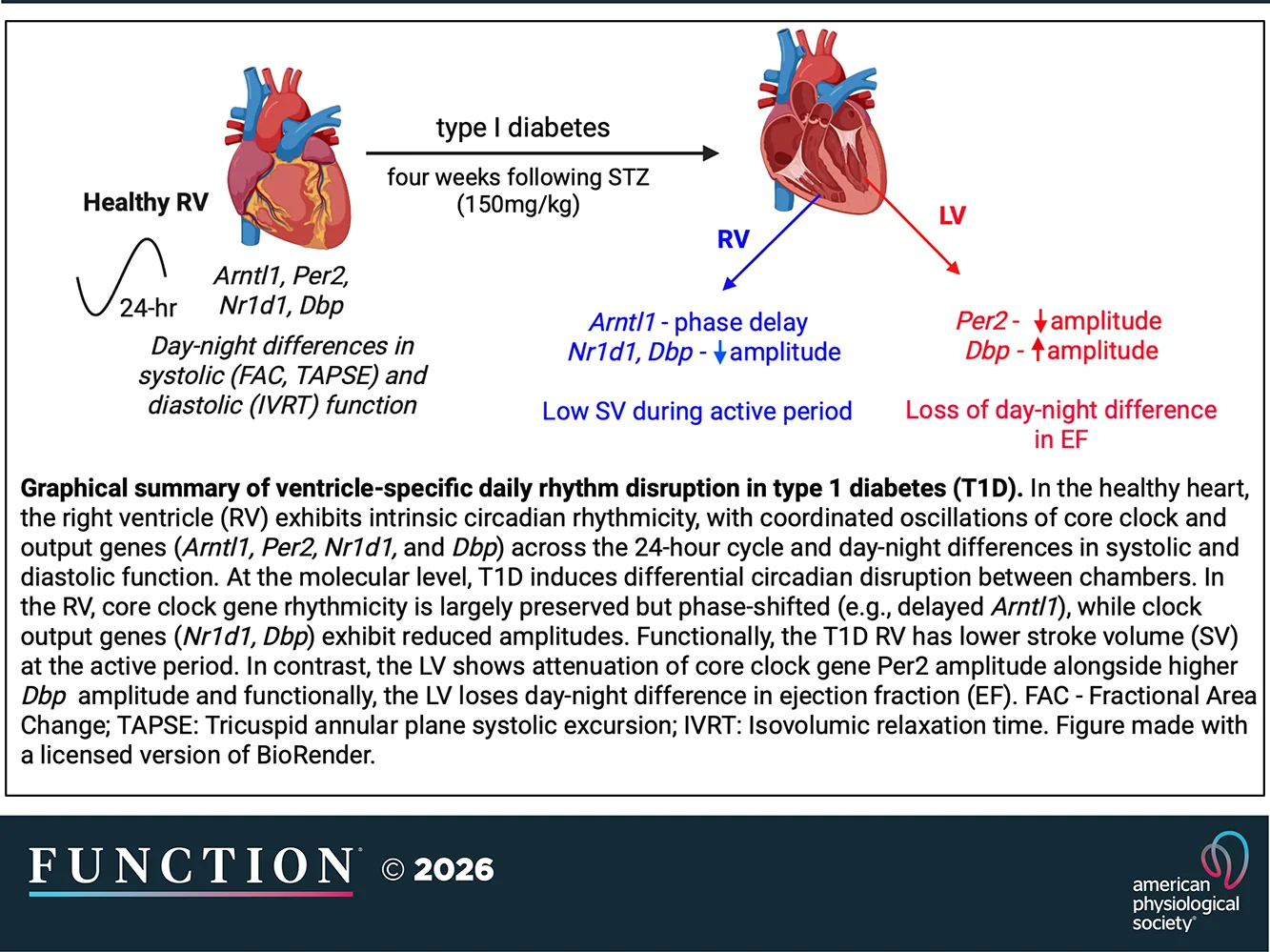

## INTRODUCTION

The circadian system comprises a central master clock in the brain and all peripheral oscillators, including throughout the cardiovascular system, which permits the cardiovascular system to anticipate and adapt to 24-h cycles. In the heart, these intrinsic rhythms regulate key functions including myocardial metabolism, contractility, and responsiveness to neurohumoral and hormonal cues such as insulin (1–3). The mammalian autonomous oscillator is composed of the basic helix-loop-helix ARNT-like protein (BMAL1*/Arntl1*) and circadian locomotor output cycles protein kaput (*Clock*), which heterodimerize and accumulate during the day to transcribe period (*Per*) and cryptochrome (*Cry*). Following transcription, *Pers* and *Crys* leave the nucleus and undergo translation in the cytoplasm to PERS and CRYS, where they form a complex that reenters the nucleus to inhibit BMAL1 and CLOCK, thereby stopping their own transcription (4, 5). This core negative feedback loop recurs about every 24 h, and the precisely controlled duration underlies the internal period length of various circadian rhythms. This molecular clock drives rhythmic expression of downstream clock output genes including nuclear receptor subfamily 1 group D member 1 (*Nr1d1*), D-box-binding PAR BZIP transcription factor (*Dbp*), and various metabolic genes including uncoupling protein 3 (*Ucp3*) and pyruvate dehydrogenase kinase 4 (*Pdk4*). Together, these and many others regulate fatty acid oxidation, mitochondrial function, and glucose utilization in a time-of-day-dependent manner (6), highlighting the pervasive role of the clock in coordinating cardiac physiology.

Emerging evidence indicates that diabetes disrupts circadian organization, though it is not yet fully clear how T1D impacts the cardiac clock. Diabetes mellitus remains a global health crisis, with type 1 diabetes (T1D) posing a persistent challenge to cardiovascular health (7). A landmark study by Young et al. (8) demonstrated that insulin-dependent diabetes phase-shifts the expression of *Bmal1*, *Per2*, *Clock*, and *Dbp* in the rat heart by ∼3 h. However, subsequent work suggested that circadian expression of *Bmal1*, *Per2*, and *Dbp* in the heart was preserved in mice with T1D despite shifted and blunted rhythms in other tissues such as the kidney and liver (9). Therefore, the impact of T1D on the cardiac clock lacks consensus, and despite these foundational observations, little is known about how diabetes impacts circadian regulation within the heart, particularly at the level of individual cardiac chambers. Most cardiovascular health and disease research has historically focused on the left ventricle (LV), based on the assumption that LV-derived mechanisms are largely translatable to the right ventricle (RV). However, this view is being increasingly challenged. Experimental and clinical studies alike reveal RV structural and functional remodeling in both type 1 and type 2 diabetes (10–12). Emerging evidence demonstrates that diabetes induces structural and functional alterations in the RV independent of and distinct from changes in the LV (13). T1D causes both RV and LV dysfunction, mediated by pathological remodeling of each ventricle, with RV diastolic dysfunction developing in a temporally distinct pattern relative to LV changes (14). However, despite the growing recognition that RV remodeling occurs with T1D, how T1D impacts circadian regulation within and between ventricular chambers is not yet known. To address this gap, we investigated expression of circadian genes and cardiac function across the 24-h cycle in both the LV and RV of a well-established mouse model of T1D.

## MATERIALS AND METHODS

### Animals

All experiments were conducted in accordance with institutional guidelines and were approved by the Institutional Animal Care Users Committee at the University of Wyoming. Experiments were conducted in male mice at ∼11 wk of age. Mice were fed standard laboratory chow (Lab Diet 5001) with water and food provided ad libitum. Mice were maintained on a 12-h light/dark cycle, with lights on at 7:00 AM (ZT0) and lights off at 7:00 PM (ZT12).

Diabetes was induced using an established streptozotocin (STZ) protocol that reliably produces T1D in mice (15). STZ (Cayman Chemical, #13104) was freshly dissolved in 0.1 M sodium citrate buffer (pH 4.5) immediately before use. Mice were fasted for 4 h before injection to enhance STZ uptake and efficacy. A single intraperitoneal injection of STZ at 150 mg/kg body wt was administered at 11 wk of age at ZT4 to achieve hyperglycemia while minimizing acute toxicity. Control animals received an equivalent volume of citrate buffer vehicle. Blood glucose was measured weekly using tail blood to validate hyperglycemia at *weeks 12*, *13*, *14*, and *15*. At study termination, animals underwent echocardiography at 16 wk of age either ZT0–4 or ZT12–16 and were humanely euthanized by pentobarbital (100 mg/kg) at ZT0, 4, 8, 12, 16, or 20. At euthanasia, the RV was dissected from the LV and septum, wet weights were measured, and ventricles were flash‐frozen and stored at −80°C.

### Echocardiography

Cardiac function was assessed via transthoracic echocardiography (Vevo 2100; FujiFilm). Mice were induced with 2.5% isoflurane in compressed air and maintained between 1% and 3% to permit a heart rate (HR) > 400 beats/min. Respiratory rate and HR were monitored with 4-lead limb ECG (FujiFilm). Parasternal short axis views of the RV were captured in B-mode and M-mode. Parasternal long axis views of the RV and pulmonary artery (PA) were captured in B-mode. Pulse wave Doppler views of the PA were captured for pulmonary acceleration times (PATs). RV M-mode tracings were used to assess RV wall thickness during systole and diastole (RVFW;s and RVFW;d). Short-axis B-mode tracings were used to assess fractional area change (FAC). RV stroke volume (SV) and cardiac output (CO) were calculated by Visual Sonics software. Two-dimensional parasternal long-axis B-mode views and parasternal short-axis B-mode and M-mode views of the LV were captured. Using VevoLab software, cine loop analysis was conducted on cardiac cycles between respirations to minimize respiratory artifact. Endocardial borders were traced in long-axis B-mode up to the aortic valve to assess total areas, volumes, ejection fraction (EF) [EF = (LVEDv − LVESv)/LVEDv × 100], fractional shortening (FS) [FS = (LVIDd − LVIDs)/LVIDd × 100], and cardiac output (CO). Short-axis M-mode tracings measured parallel to the axis of the papillary muscles were used to assess anterior and posterior LV wall thickness during diastole and systole. All echocardiography was performed either between ZT0–4 or ZT12–16.

Echocardiography data were analyzed by two-way ANOVA (condition × ZT) with post hoc Student’s *t* test to compare within condition and ZT differences. Significance was set a priori at α < 0.05. *P < 0.05 ZT0 versus ZT12 within group, *^*P* < 0.05 interaction of ZT and condition as assessed by two-way ANOVA. Statistical tests were performed using Prism Version 9 (GraphPad). Data are expressed as means ± SEM.

### Real-Time RT-PCR

RNA was extracted from the LV and RV using standard Trizol protocols and reverse-transcribed using iScript cDNA synthesis kit (ThermoFisher). Real-time RT-PCR was performed using SYBR Green Supermix (ThermoFisher). Data were normalized to the housekeeping gene ribosomal protein 26 (*Rpl26*). Delta delta Ct values were calculated within condition, and data were expressed as fold change relative to ZT0. Oligonucleotide sequences were as follows: *Arntl1* (forward: 5′- ATCAGCGACTTCATGTCTCC-3'; reverse: 5′- CTCCCTTGCATTCTTGATCC-3′), *Per2* (forward: 5′- GCCAAGTTTGTGGAGTTCCTG-3′; reverse: 5′- CTTGCACCTTGACCAGGTAGG-3′), *Rpl26* (forward: 5′- CGAGTCCAGCGAGAGAAGG-3′; reverse: 5′- GCAGTCTTTAATGAAAGCCGTG-3′), *Nr1d1* (forward: 5′- TCCCAGGCTTCCGTGACCTTT-3′; reverse: 5′- TTGTGCGGCTCAGGAACATCA-3′), *Pdk4* (forward: 5′- TGAACACTCCTTCGGTGCAG-3′; reverse: 5′- TGTCTACAAACTCTGACAGGGC-3′), *Dbp* (forward: 5′- CACCGTGGAGGTGCTAATGA-3′; reverse: 5′- CATGGCCTGGAATGCTTGA-3′), *Ucp3* (forward 5′- ACAAAGGATTTGTGCCCTCCT-3′; reverse: 5′- AAAACGGAGATTCCCGCAGT-3′).

### Cosinor Analysis

To assess rhythmicity of clock gene expression, a cosinor regression approach was applied to gene expression data sampled across six Zeitgeber times (ZT0, ZT4, ZT8, ZT12, ZT16, and ZT20). For each gene, a single-component cosinor model was fitted using ordinary least squares (OLS) regression with cosine and sine terms representing a 24-h period of the form:

$$Y=\;\text{mesor }+\;\text{A}\times \text{cos}\left(2\pi t/24\right)\;+\;\text{B}\times \text{sin}\left(2\pi t/24\right)\;+\;\varepsilon$$

where *Y* is the gene expression value, mesor is the intercept, *t* is the Zeitgeber time, and ε is the residual error term. Amplitude and acrophase were derived from the fitted coefficients A and B as amplitude = √(A^2^ + B^2^) and acrophase = atan2(B, A) × 24/2π, expressed in hours modulo 24. To compare rhythms between control (Con) and T1D, an interaction cosinor model was fit that included condition as a categorical predictor along with its interactions with the cosine and sine terms:

$$Y=\;\text{mesor }+\;\text{A}\times \text{cos}\left(2\pi t/24\right)\;+\;\text{B}\times \text{sin}\left(2\pi t/24\right)\;+\;\beta \times \text{condition }+\;\text{A}\prime \times \left(\text{cos }\times \;\text{condition}\right)\;+\;\text{B}\prime \times \left(\text{sin }\times \;\text{condition}\right)\;+\;\varepsilon$$

Two Wald tests were derived from this full interaction model. First, a rhythm *P* value was calculated by jointly testing all cosine and sine terms, including interaction terms, reflecting whether significant 24-h rhythmicity was present across either condition. Second, a condition difference *P* value was calculated by jointly testing only the interaction terms (cos × condition and sin × condition), reflecting whether the fitted 24-h waveform differed between Con and T1D. For genes exhibiting significant rhythmicity, mesor, amplitude, and acrophase were then compared between conditions. Because amplitude and acrophase are nonlinear transformations of the fitted coefficients, *P* values for differences in these parameters were estimated using the delta method, which uses a Taylor series approximation and the model covariance matrix to estimate standard errors of parameter differences. All analyses were performed in Python 3.11.8 using OLS regression implemented in statsmodels 0.14.0. Figures were generated using matplotlib 3.10.0, with individual data points overlaid on fitted cosinor curves for each condition.

## RESULTS

**Figure 1.**
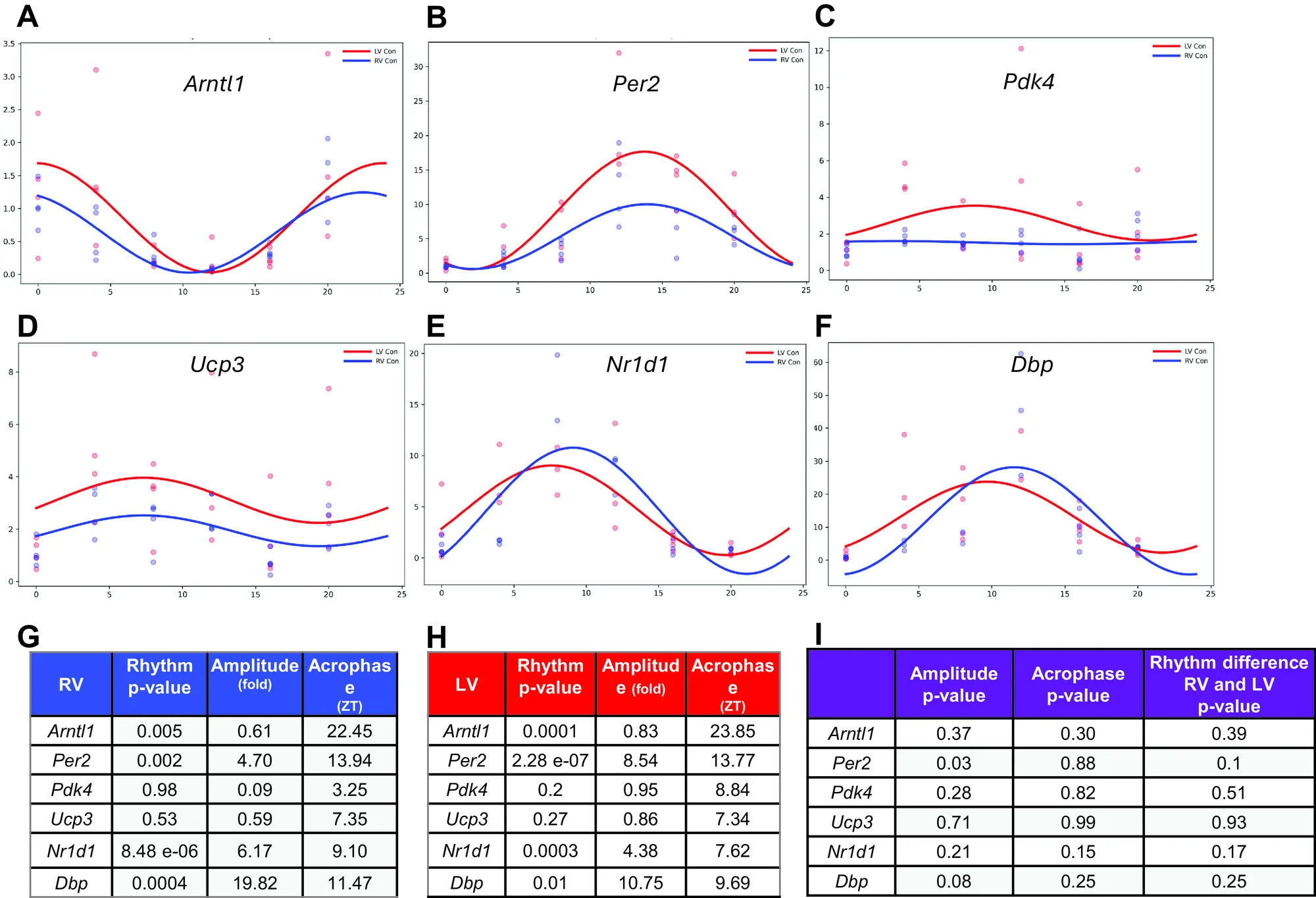
Ventricle-specific circadian transcriptional rhythms in the healthy right ventricle (RV) and left ventricle (LV). Cosinor curves of core clock, clock-controlled output genes, and metabolic gene expression across the 24-h light-dark cycle in the left ventricle (LV) and right ventricle (RV) of control male mice. Transcript levels of *Arntl1* (*A*), *Per2* (*B*), *Pdk4* (*C*), *Ucp3* (*D*), *Nr1d1* (*E*), and *Dbp* (*F*) were measured by qRT-PCR at 6 zeitgeber time (ZT0, ZT4, ZT8, ZT12, ZT18, ZT22) and normalized to the reference gene *Rpl26*. The *x*-axis represents zeitgeber time (ZT; hours), where ZT0 denotes lights-on and ZT12 denotes lights-off. *G* and *H*: summary table of cosinor-derived parameters for each gene in the RV and LV. Amplitude (half the peak-to-trough range) and acrophase (clock time of peak expression, in ZT hours). *I*: cosinor analysis comparing LV vs. RV rhythms for each transcript. Shown are *P* values for between-chamber differences in amplitude, acrophase, and overall rhythm waveform (*F*-test). LV—red and RV—blue. *n* = 4 per group/ZT.

To determine whether the healthy RV exhibits daily rhythmicity, we performed cosinor analysis on six circadian transcripts, *Arntl1*, *Per2*, *Pdk4*, *Ucp3*, *Nr1d1*, and *Dbp* in the LV and RV of control mice (Fig. 1, *A*–*F*). The RV demonstrated significant 24-h rhythmicity for *Arntl1*, *Per2*, *Nr1d1*, and *Dbp*, with cosinor parameters summarized in Fig. 1*G*. The presence of rhythmic expression in the RV, including the expected temporal ordering of positive- and negative-limb clock gene peaks, indicates that the healthy RV clock operates with similar temporal organization between the RV and LV. Indeed, in the LV, overall 24-h rhythmicity was detected for *Arntl1*, *Per2*, *Nr1d1*, and *Dbp* (Fig. 1, *A*–*F*), consistent with prior reports (3, 16). Cosinor parameters for the LV are summarized in Fig. 1*H*. To assess whether healthy LV and RV differed in the properties of their circadian oscillations, we compared rhythmic waveforms, amplitude, and acrophase for each transcript between LV and RV (Fig. 1*I*). No significant interchamber differences in rhythmic waveform were detected for any of the six genes, except for *Per2* amplitude, which was lower in the RV compared with the LV, suggesting that under healthy conditions, the RV and LV share broadly conserved circadian timing and rhythmic amplitude.

To understand how T1D shifts RV and LV clocks, we first validated the model of STZ as confirmed by sustained hyperglycemia (blood glucose > 300 mg/dL) throughout the study period (Supplemental Fig. S1*A*). As expected, T1D animals had lower body weight compared with controls (Supplemental Fig. S1*C*), with a nadir at ZT12 that was most pronounced relative to Con at ZT12 and ZT16 (Supplemental Fig. S1*B*).

**Figure 2.**
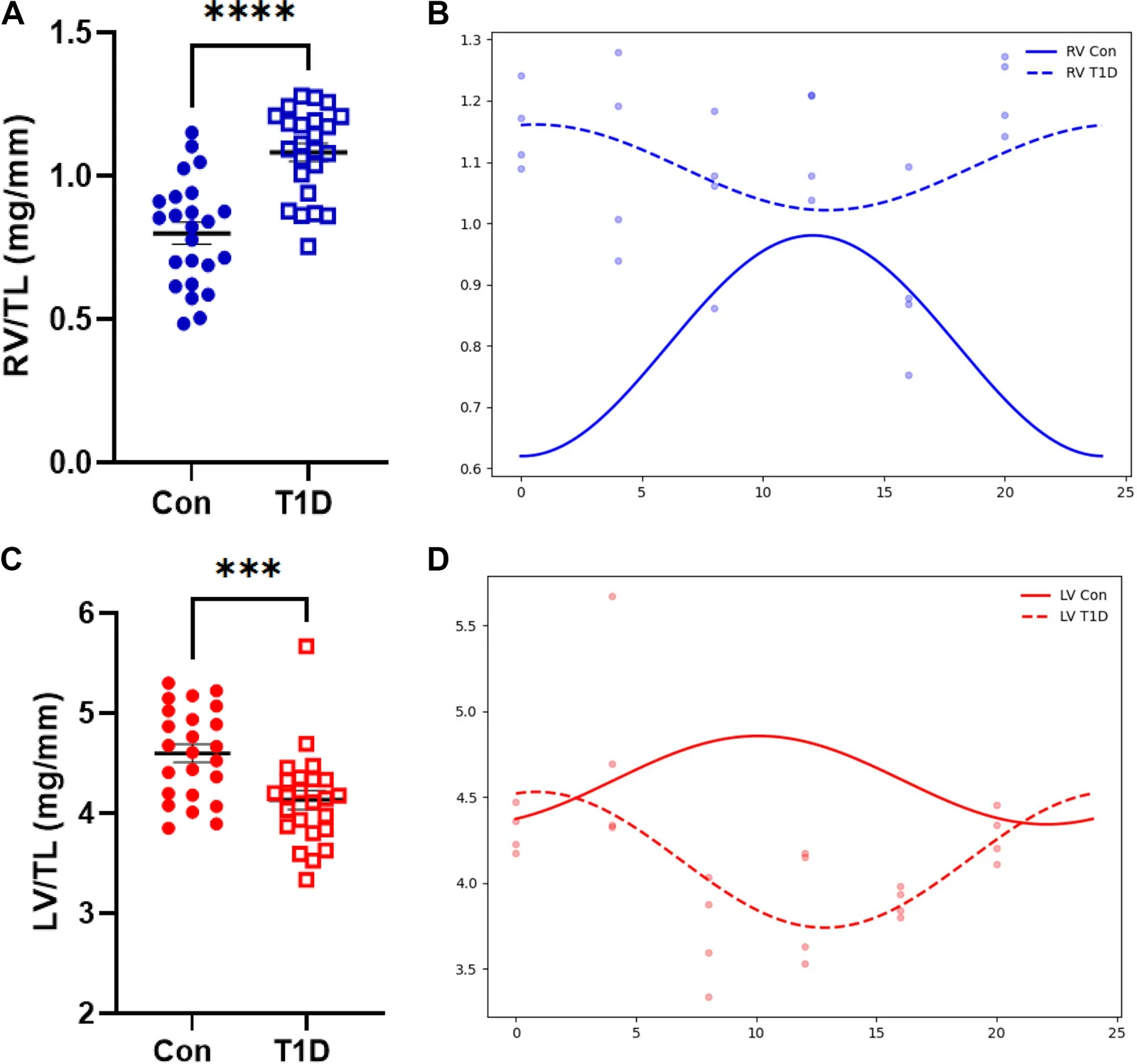
T1D differentially impacts left and right ventricular mass across the 24-h cycle. *A*: right ventricular mass normalized to tibia length (RV/TL, mg/mm) pooled from all 6 zeitgeber time (ZT). T1D mice show significantly higher RV/TL compared with controls (Con). *B*: cosinor analysis fit for RV/TL demonstrating altered circadian oscillations in T1D compared with controls. *C*: left ventricular mass normalized to tibia length (LV/TL, mg/mm). T1D mice show significantly lower LV/TL compared with controls. *D*: cosinor analysis fit for LV/TL demonstrating altered circadian oscillations in T1D compared with controls. Data are expressed as means ± SEM. *n* = 4 per group/ZT. ***P < 0.001, ****P < 0.0001 by Student's *t* test.

To determine whether T1D impacts cardiac mass in a chamber-specific and time-dependent manner, we measured RV and LV weight normalized to tibia length (RV/TL and LV/TL). T1D animals had greater RV/TL than Con (Fig. 2*A*). Cosinor analysis revealed a robust 24-h rhythm in RV/TL in control animals, with a sinusoidal waveform peaking around ZT12. In contrast, T1D animals showed a markedly elevated and comparatively attenuated cosinor fit across the 24-h cycle, suggesting that T1D disrupts daily rhythms in RV mass (Fig. 2*B*). LV/TL was reduced in T1D animals compared with Con (Fig. 2*C*). Cosinor analysis of LV/TL revealed a modest but visible rhythm in Con animals, whereas T1D animals showed a flattened and reduced waveform with lower overall mass across the cycle (Fig. 2*D*). Together, these data demonstrate that T1D produces opposing chamber-specific effects on ventricular mass; RV hypertrophy and LV atrophy each with a distinct alteration in the 24-h pattern.

**Figure 3.**
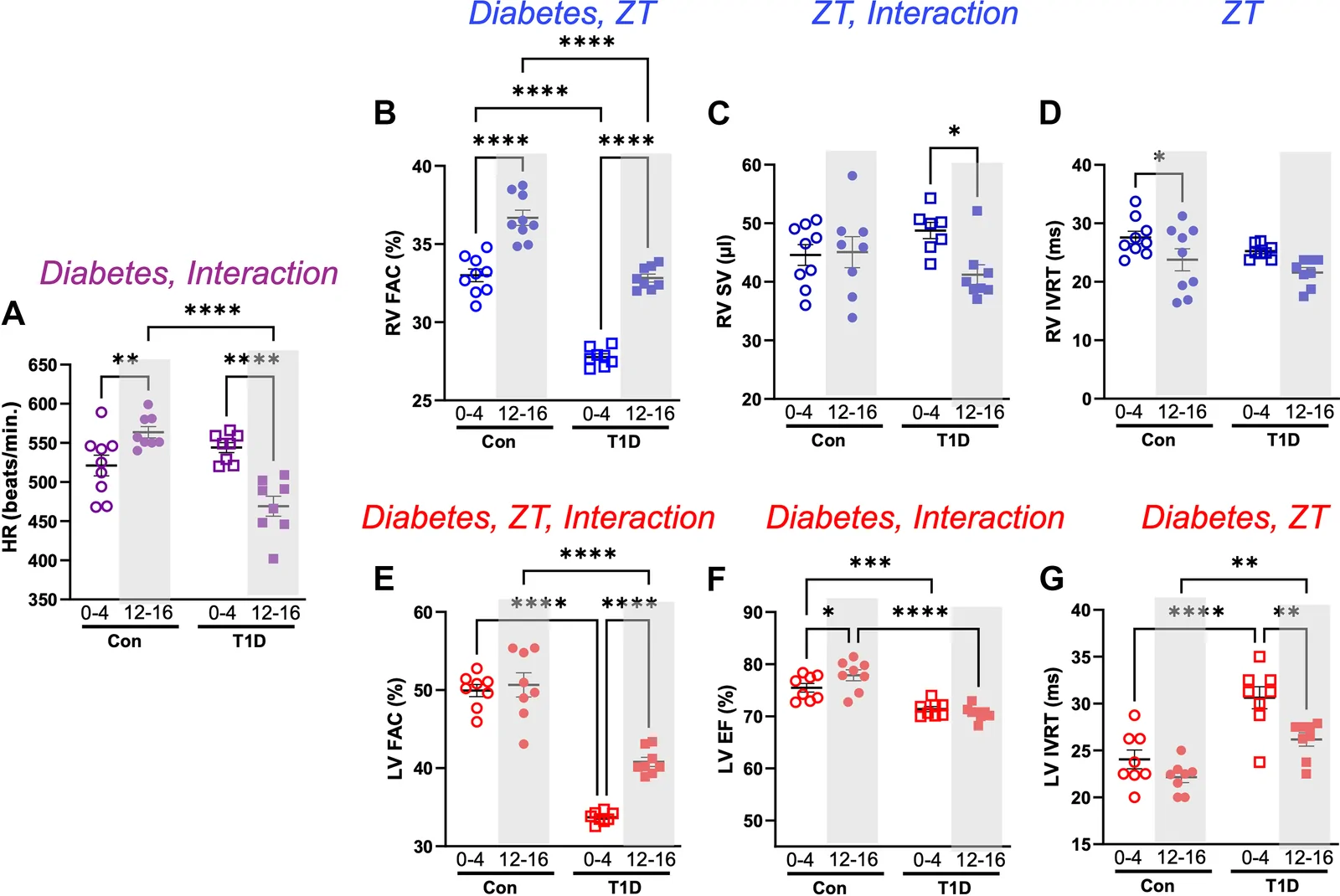
Echocardiography in Con and T1D at ZT0–4 and ZT12–16 suggests that T1D imposes an aberrant time-of-day variation in right ventricle (RV) and left ventricle (LV) systolic function. *A*: in control mice, heart rate (HR) was higher at ZT0–4 compared with ZT12–16, whereas T1D animals showed the opposite pattern, with HR declining from ZT0–4 to ZT12–16. *B*: RV fractional area change (RV FAC) was reduced by T1D with T1D animals showing lower RV FAC at both ZT compared with controls. *C*: RV stroke volume (SV) was lower at ZT12–16 compared with ZT0–4 in T1D animals. *D*: RV isovolumetric relaxation time (RV IVRT) showed a main effect of ZT, with values declining from ZT0–4 to ZT12–16. *E*: LV FAC was reduced in T1D animals compared with controls, with a significant diabetes × ZT interaction. *F*: LV ejection fraction (LV EF) was reduced in T1D at both ZT, with T1D animals losing the day-night difference in EF. *G*: LV IVRT was prolonged with T1D, irrespective of ZT. Data were analyzed by 2-way ANOVA (ZT × condition). Data are expressed as means ± SEM. *n =* 8 per group. *P < 0.05, **P < 0.01, ***P < 0.001, ****P < 0.0001. ZT, zeitgeber time.

Echocardiographic assessment was performed between ZT0–4 and ZT12–16 under anesthesia and is summarized in Table 1. Heart rate (HR) was affected by diabetes and the diabetes × ZT interaction was significant. In Con mice, as expected, HR was higher at ZT12–16 compared with ZT0–4. However, T1D animals showed the opposite pattern, with HR declining from ZT0–4 to ZT12–16 (Fig. 3*A*), suggesting loss of circadian control of HR. RV fractional area change (RV FAC) was reduced by T1D with T1D animals showing lower RV FAC at both ZT compared with Con (Fig. 3*B*). RV stroke volume (SV) showed a significant diabetes × ZT interaction and was lower at ZT12–16 compared with ZT0–4 in T1D (Fig. 3*C*); thus T1D results in low RV SV during the active period. RV isovolumetric relaxation time (RV IVRT) showed a main effect of ZT, with values declining from ZT0–4 to ZT12–16 consistent with improved relaxation during the active period (Fig. 3*D*). LV FAC was reduced in T1D compared with Con at both ZT and also showed a significant diabetes × ZT interaction, with FAC higher at ZT12–16 compared with ZT0–4 in T1D animals (Fig. 3*F*). LV ejection fraction (LV EF) was lower in T1D animals at both ZT (Fig. 3*E*), and mice with T1D lost the time-of-day difference in EF. LV IVRT was shorter during the active period ZT12–16, and T1D animals exhibited prolonged LV IVRT compared with Con (Fig. 3*G*). Thus, T1D impairs day-night LV systolic (FAC, EF) function.

**Table 1.** Summary of RV (top) and LV (bottom) echocardiography in control (Con) and type 1 diabetes (T1D) male mice at ZT0–4 and ZT12–16

	Con ZT0–4	Con ZT12–16	T1D ZT0–4	T1D ZT12–16	2-Way ANOVA
HR, beats/min	521 ± 13	549 ± 16	533 ± 7	469 ± 13	Diabetes, interaction
RV FAC, %	33 ± 0.4	37 ± 0.5	28 ± 0.2	33 ± 0.2	Diabetes, ZT
TAPSE, mm	0.53 ± 0.01	0.57 ± 0.02	0.54 ± 0.01	0.63 ± 0.01	ZT
RV SV, µL	44 ± 1.8	45 ± 2.5	45 ± 3.3	41 ± 1.6	ZT, interaction
E/A	0.55 ± 0.03	0.69 ± 0.04	0.64 ± 0.01	0.71 ± 0.01	ns
E/e′	10.7 ± 1.0	12.1 ± 1.4	12.0 ± 0.3	13.0 ± 1.0	ZT
IVRT, ms	28 ± 1.0	24 ± 1.9	25 ± 0.4	22 ± 0.8	ZT
RVAWs, mm	0.60 ± 0.02	0.57 ± 0.04	0.63 ± 0.01	0.66 ± 0.00	Diabetes
RVAWd, mm	0.33 ± 0.01	0.36 ± 0.03	0.44 ± 0.01	0.47 ± 0.01	Diabetes
Area;s, mm^2^	5.6 ± 0.34	5.6 ± 0.22	3.6 ± 0.12	4.5 ± 0.12	Diabetes
Area;d, mm^2^	8.7 ± 0.57	8.8 ± 0.34	5.0 ± 0.13	6.9 ± 0.17	Diabetes
PAT, ms	18.3 ± 1.0	15 ± 1.0	19.1 ± 1.2	18.0 ± 0.6	ZT
ET, ms	44 ± 1.6	40 ± 2.4	50 ± 1.1	36 ± 1.1	ZT, interaction
LV EF, %	75 ± 0.8	78 ± 1	71 ± 0.8	71 ± 0.6	Diabetes, interaction
LV FAC, %	50 ± 0.7	51 ± 1.5	34 ± 0.4	41 ± 0.6	Diabetes, ZT, interaction
LV FS, %	45 ± 1	42 ± 1.7	30 ± 2.4	31 ± 0.8	Diabetes
MAPSE, mm	0.61 ± 0.02	0.60 ± 0.02	0.55 ± 0.03	0.65 ± 0.01	ns
LV SV, µL	33 ± 2	34 ± 1	39 ± 3	40 ± 3	Diabetes, ZT
E/A	1.4 ± 0.05	1.5 ± 0.06	1.3 ± 0.05	1.2 ± 0.04	Diabetes, ZT, interaction
E/e′	35 ± 2.7	22 ± 2.5	33 ± 3.9	28 ± 0.36	Diabetes, interaction
IVRT, ms	24 ± 0.9	22 ± 0.6	31 ± 1.9	26 ± 1.5	Diabetes, ZT
LVAWs, mm	1.47 ± 0.04	1.47 ± 0.07	1.21 ± 0.1	1.2 ± 0.05	Diabetes
LVAWd, mm	0.90 ± 0.04	0.91 ± 0.05	0.77 ± 0.06	0.78 ± 0.05	Diabetes
Area;s, mm^2^	5.5 ± 0.4	5.2 ± 0.3	6.4 ± 0.9	6.4 ± 0.6	Diabetes
Area;d, mm^2^	10.8 ± 0.8	9.7 ± 0.4	9.7 ± 1.3	10.8 ± 1	Diabetes, ZT, interaction
AAT, ms	17.5 ± 0.9	15.9 ± 0.7	19.0 ± 0.9	17.8 ± 1.6	Diabetes, ZT
ET, ms	45 ± 1.2	38 ± 0.5	44 ± 1.4	40 ± 0.6	ZT

Data are expressed as mean ± SEM. *n* = 8 per group. Results from 2-way ANOVA (ZT × condition) with *P* < 0.05 are provided. ns, not significant. AAT, aortic acceleration time; AWs and Awd, anterior wall thickness during systole and diastole; area, area during systole and diastole; E/A, early/atrial filling; E/e′, ratio of early diastolic mitral inflow velocity (E) to early diastolic mitral annular tissue velocity (e′); EF, ejection fraction; ET, ejection time; FAC, fractional area change; FS, fractional shortening; HR, heart rate; IVRT, isovolumic relaxation time; MAPSE, mitral annular plane systolic excursion; PAT, pulmonary acceleration time; SV, stroke volume; TAPSE, tricuspid annular plane systolic excursion; ZT, zeitgeber time.

**Figure 4.**
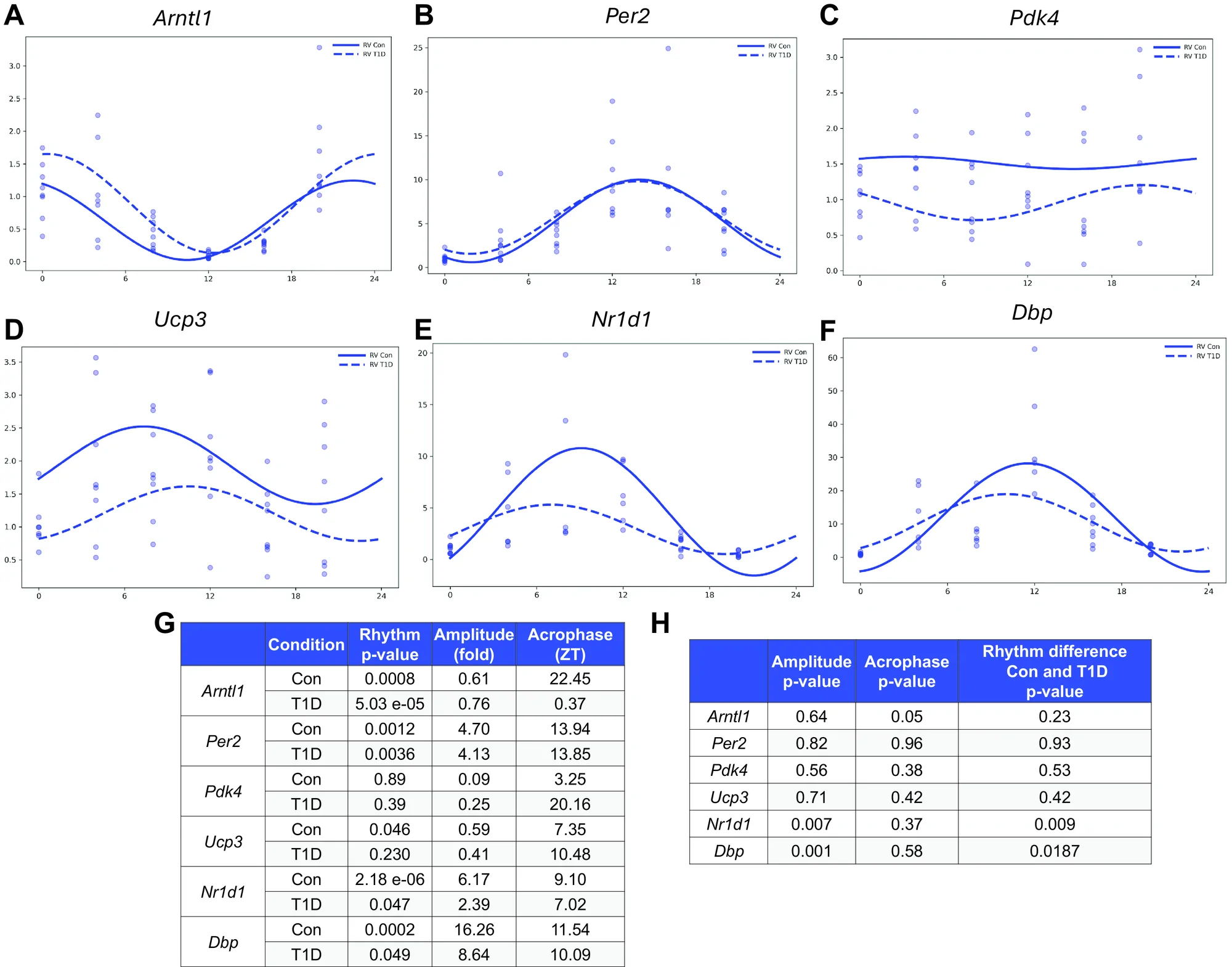
Alterations in gene expression rhythms in the T1D right ventricle (RV). *A*: *Arntl1* exhibited 24-h rhythmicity in both Con and T1D animals with a delay in the acrophase in T1D mice. *B*: *Per2* was robustly rhythmic with no condition difference and a highly conserved acrophase. *C* and *D*: the metabolic clock targets *Pdk4* and *Ucp3* did not achieve significant rhythmicity in either condition and showed no condition difference. *E*: *Nr1d1* exhibited significant rhythmicity in Con, and the T1D waveform was different from Con, with an advanced acrophase. *F*: *Dbp* showed rhythmicity in Con, with T1D waveform significantly different. Amplitude was lower, whereas acrophase was preserved. *G*: table showing *P* values for rhythmicity (suggesting if there is presence of a significant 24-h oscillation), amplitude, and acrophase in Con and T1D. *H*: *P* values for amplitude, acrophase, and rhythm difference between Con and T1D. Expression was normalized to *Rpl26* and expressed as fold change relative to within-group ZT0. RV Con—blue solid line and RV T1D—blue dashed line. *n* = 4 per group/ZT. ZT, zeitgeber time.

To determine whether T1D disrupts cardiac circadian clock gene expression, we measured the rhythmic transcripts *Arntl1*, *Per2*, *Nr1d1*, *Dbp*, *Pdk4*, and *Ucp3* in the RV of Con and T1D mice (Fig. 4, *A*–*F*), with cosinor parameters for each condition summarized in Fig. 4*G*. Cosinor analysis revealed selective alterations in 24-h rhythmic gene expression in T1D. *Arntl1* exhibited significant 24-h rhythmicity in both Con and T1D (Fig. 4, *A* and *G*); however, the T1D acrophase was delayed relative to Con, shifting from ZT22.5 in Con to ZT0.4 in T1D, a delay of ∼2 h. No significant differences in amplitude or overall rhythmicity were detected. *Per2* was robustly rhythmic under both Con and T1D with a conserved acrophase (∼ZT13.8) and no significant differences in amplitude, acrophase, or overall rhythmicity (Fig. 4, *B*, *G*, and *H*). *Pdk4* did not achieve significant rhythmicity in either condition and showed no effect of T1D (Fig. 4, *C*, *G*, and *H*). The metabolic gene *Ucp3* was rhythmic in Con but lost statistical rhythmicity in T1D (Fig. 4, *D* and *G*); however, no between-condition differences in amplitude or acrophase were detected (Fig. 4, *G* and *H*). In contrast, the clock output genes *Nr1d1* and *Dbp* exhibited more pronounced changes in rhythmic expression. *Nr1d1* was rhythmic in Con and T1D, but T1D was associated with a reduced amplitude (6.17 in Con vs. 2.39 in T1D) and a difference in rhythmic waveform between conditions (Fig. 4, *E*, *G*, and *H*). Similarly, *Dbp* was rhythmic in Con and T1D, with a reduced amplitude (16.26 in Con vs. 8.64 in T1D) in T1D and a significant difference in the overall fitted rhythm between conditions (Fig. 4, *F*, *G*, and *H*). Together, these data indicate that T1D selectively attenuates the amplitude of *Nr1d1* and *Dbp* rhythmic expression in the RV and delays the phase of *Arntl1* while largely preserving the rhythmicity and phase of the core clock component *Per2*.

**Figure 5.**
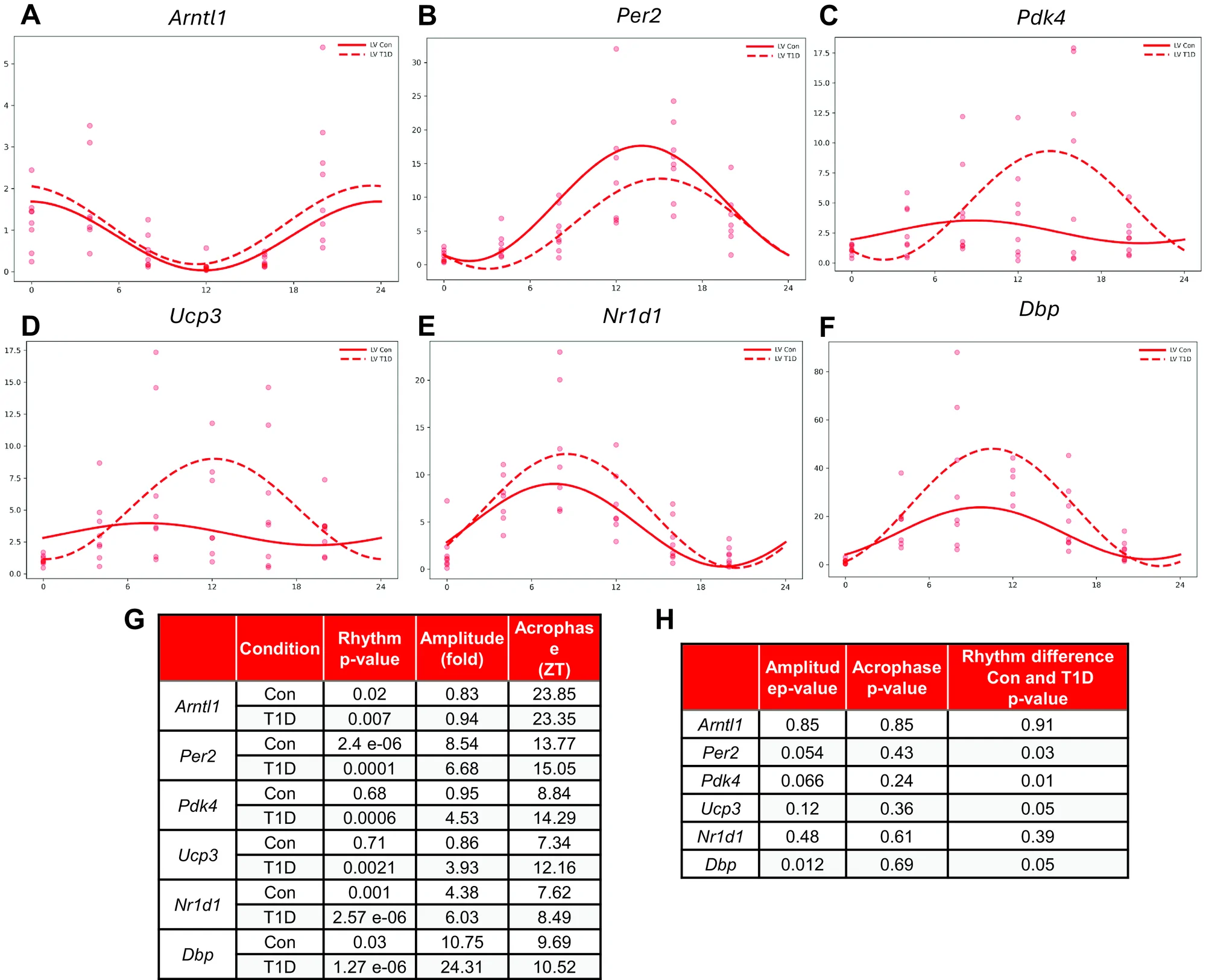
Alterations in gene expression rhythms in the T1D left ventricle (LV). *A*: *Arntl1* exhibited 24-h rhythmicity in both Con and T1D animals with no significant differences in amplitude and acrophase in T1D mice. *B*: *Per2* was robustly rhythmic in both groups with significant condition differences and reduced amplitude. *C* and *D*: the metabolic clock targets *Pdk4* and *Ucp3* did not achieve significant rhythmicity in either condition. *E*: *Nr1d1* was rhythmic in Con and T1D, with T1D showing no changes in amplitude and acrophase. *F*: *Dbp* showed rhythmicity in Con and T1D, with T1D showing a near-tripling of amplitude, with acrophase preserved. *G*: table showing *P* values for rhythmicity (suggesting if there is presence of a significant 24-h oscillation), amplitude, and acrophase in Con and T1D. *H*: *P* values for amplitude, acrophase, and rhythm difference between Con and T1D. Expression was normalized to *Rpl26* and expressed as fold change relative to within-group ZT0. LV Con—red solid line and LV T1D—red dashed line. *n* = 4 per group/ZT. ZT, zeitgeber time.

LV gene expression in T1D showed a pattern distinct from and in several respects opposite to that observed in the RV (Fig. 5, *A*–*F*), with cosinor parameters summarized in Fig. 5*G*. *Arntl1* was rhythmic in both Con and T1D, with no significant differences in amplitude, acrophase, or rhythmic waveform between conditions (Fig. 5, *A*, *G*, and *H*). *Per2* was robustly rhythmic under both conditions; however, T1D was associated with a reduced amplitude (8.54 in Con vs. 6.68 in T1D) and a difference in overall rhythmicity between conditions (Fig. 5, *B*, *G*, and *H*). *Pdk4* and *Ucp3* gained rhythmicity in the LV under T1D, with a significant difference in fitted rhythmic waveforms between conditions for both transcripts, though the amplitudes and acrophases did not reach statistical significance (Fig. 5, *C*, *D*, *G*, and *H*). *Nr1d1* was rhythmic in both Con and T1D (Fig. 5, *E*, *G*, and *H*). *Dbp* was rhythmic in both conditions, but T1D was associated with a higher amplitude (10.75 in Con vs. 24.31 in T1D) and a significant difference in overall rhythmicity between conditions, with acrophase largely preserved (Fig. 5, *F*, *G*, and *H*). Together, these findings reveal that T1D amplifies rather than attenuates daily rhythms in the LV, a response directionally opposite to the rhythmic gene expression patterns observed in the RV.

## DISCUSSION

The RV has long been understudied in the context of diabetic cardiomyopathy, often overshadowed by the well-documented effects of T1D on LV structure and function. Yet RV dysfunction is a strong independent predictor of patient outcomes (17) and the two ventricles differ fundamentally in many physiological and pathological contexts. Whether T1D differentially alters the RV and LV circadian clocks and the functional consequences of those differences has not been investigated. We demonstrate for the first time that T1D exerts divergent, chamber-specific effects on cardiac circadian regulation, function, and molecular clock gene expression. Our findings reveal that T1D does not suppress the cardiac clock uniformly, but rather differentially remodels daily rhythms in a ventricle-specific manner. Understanding how and why the RV and LV are differentially affected by T1D opens new avenues for chamber-targeted chronotherapeutic strategies and for improving the timing of cardiovascular risk assessment in patients with diabetes.

A foundational and novel observation of the present study is the demonstration that the healthy mammalian RV harbors robust, 24-h transcriptional rhythms. To our knowledge, this is the first report to characterize circadian clock gene oscillations in the RV and report time-of-day dependence of RV function, establishing it as a likely peripheral circadian oscillator. Prior investigations of the cardiac circadian clock have focused almost exclusively on the LV or undifferentiated whole heart preparations (3, 16), leaving the RV a chronobiological blind spot despite its critical roles in healthy cardiopulmonary perfusion and cardiopulmonary disease. Echocardiographic analysis showed significant day-night differences in RV FAC, with higher FAC during the dark/active phase (ZT12–16) than the light phase (ZT0–4). RV diastolic function also differed by time of day, with faster rates of relaxation as measured by IVRT during the dark/active phase (ZT12–16). Our cosinor analysis demonstrated that *Arntl1*, *Per2*, *Nr1d1*, and *Dbp* all oscillate with significant 24-h periodicity in the RV of control male mice, with acrophase timings that are temporally coherent with the canonical transcription-translation feedback loop hierarchy. Specifically, *Per2* and *Dbp* peak during the mid-to-late light phase (ZT13.9 and 11.5, respectively), whereas *Arntl1* peaks near the dark onset (ZT22.5), mirroring the antiphase relationship that defines a functional circadian oscillator. This temporal architecture in the RV is strikingly similar to that observed in the LV (3) and in other peripheral clocks such as liver (18) and skeletal muscle (19), suggesting that the RV has robust circadian rhythms. The identification of a functional RV clock opens an entirely new area of inquiry: whether chamber-specific clock timing has physiological relevance for right-sided cardiac function and susceptibility to RV diseases.

One of the most striking functional findings of the present work was the reversal of the diurnal HR pattern in T1D mice. In control mice, HR was higher at ZT0–4 compared with ZT12–16, reflecting the heightened sympathetic activity expected during the active dark phase in nocturnal rodents. T1D mice, however, showed completely opposite HR during day/night, with higher HR at ZT0–4 and lower HR at ZT12–16. Cardiac autonomic neuropathy is a well-documented complication of STZ-induced T1D, characterized by reduced sympathetic and parasympathetic cardiac tone and depressed HR variability (20). Patients with T1D show dampened or abolished circadian oscillations of HR variability with relative sympathetic prevalence during the night (21). A study in STZ-treated mice demonstrated reduced HR across the 24-h cycle and impaired sympathetic control of HR compared with healthy animals (22). The inversion we observed may therefore represent a severe manifestation of sympathetic circadian dysregulation compared with previously reported dampening of rhythms. The loss of circadian modulation of autonomic control has significant consequences for patients with diabetes. The loss of normal circadian autonomic tone has been proposed to explain the lack of circadian distribution of cardiac events in patients with diabetes, effectively creating continuous susceptibility to arrhythmic and thrombotic events throughout the day (23), highlighting the significance of impaired circadian HR control in the setting of diabetes.

T1D drove divergent structural and functional remodeling across the two ventricular chambers, with the functional consequences pronounced and temporally complex. While investigating the differences between the RV and LV in the context of T1D, the observation that T1D increased RV/TL while reducing LV/TL represents a chamber-specific structural divergence. LV atrophy in the STZ model is consistent with prior reports of reduced LV mass associated with insulin deficiency and loss of insulin-driven anabolic signaling in the diabetic heart (24). Previous reports have also demonstrated RV hypertrophy in mice with T1D (14). Although systemic insulin deficiency plausibly explains the loss of anabolic, growth-promoting signaling responsible for LV atrophy, the RV hypertrophy we observe is unlikely to be explained by the same systemic factor and instead more likely reflects chamber-specific loading conditions. Specifically, T1D is associated with elevated pulmonary vascular resistance and pulmonary microvascular disease (25), which would selectively increase RV afterload independent of circulating insulin levels. The RV, unlike the LV, is exquisitely sensitive to afterload, with even modest increases in pulmonary pressure causing afterload stress (26). In addition, recent work has shown that diabetes-induced RV remodeling can proceed distinct mechanisms from the LV and via different temporal resolution (13, 14, 27), providing a chamber-intrinsic metabolic mechanism that would not depend on systemic insulin signaling in the same way. Indeed, we report that functionally, both chambers exhibited systolic impairment with T1D, but with distinct temporal characteristics. In the RV, FAC was reduced at both ZT with no time-of-day variation. RV SV was lower during the active period in mice with T1D. This finding is physiologically meaningful given that the active phase (ZT12–16) in nocturnal mice corresponds to the period of peak locomotor demand, heightened cardiac output, and elevated heart rate (28), the interval during which the RV is called upon to maximally augment pulmonary blood flow. The selective attenuation of RV SV at this time of day during the high-demand window suggests that T1D impairs the RV capacity to meet circulatory demands, a deficit that would be hemodynamically consequential during the active period. In contrast, the healthy LV demonstrated clear time-of-day-dependent systolic dysfunction, with loss of the day-night variation in EF, though EF was not lower during the active period. Thus, we suggest distinct, chamber-specific mechanisms for RV hypertrophy and LV atrophy, rather than implying a single common systemic cause. Determining the relative contribution of hemodynamic afterload versus local metabolic signaling in the RV is an important question for future study.

Having established that T1D imposes ventricle-specific and temporally distinct remodeling, we next examined whether these differences were accompanied by corresponding divergence in the molecular circadian gene expression governing each ventricle. We show that in the RV, *Arntl1* remained rhythmic in T1D, but the acrophase had a phase advance of ∼2 h, whereas *Per2* was preserved in amplitude, acrophase, and rhythmicity. This selective delay of *Arntl1* without disruption of *Per2* is intriguing mechanistically, as the two genes are components of the same feedback loop and BMAL1 drives *Per2* transcription, which then feeds back to suppress the CLOCK/BMAL1 heterodimer complex. A delay in *Arntl1* acrophase without a corresponding delay in *Per2* may reflect an uncoupling within the core loop itself. In contrast to the preserved core clock, the clock output layer consisting of *Nr1d1* and *Dbp* exhibited substantial dampening of rhythmic amplitude and altered waveform structure in T1D. This distinction between an intact core oscillator and a disrupted output layer is important because the clock output genes serve as critical mediators linking the core clock to downstream metabolic and transcriptional programs. *Dbp* drives downstream clock-controlled gene expression through D-box elements and has been reported to be reduced in adipose tissue of patients with diabetes (29). *Nr1d1* is a member of a family of nuclear receptors that stabilize the molecular clock feedback loop while directly regulating cardiac metabolic gene networks (30). Cardiomyocyte-specific deletion of *Nr1d1* causes dilated cardiomyopathy and premature death (31), highlighting the critical role for these clock regulators in cardiac function. The combined loss of amplitude of both *Dbp* and *Nr1d1* in the T1D RV effectively decouples the metabolic output layer from an intact core oscillator, likely impairing the ability of the RV to anticipate and adapt to the cyclically changing metabolic demands. Studies from cardiac loss-of-clock-function models support a causal relationship between clock gene disruption and impaired cardiac structure/function, lending plausibility to our proposed observation that the dampened *Nr1d1/Dbp* rhythms in the T1D RV contribute mechanistically to the functional deficits, rather than being a coincidental downstream consequence of dysfunction. Together, our data imply that the RV clock is still “ticking,” but its output clock might be severely compromised. Notably, although cardiomyocyte-specific clock gene disruption models have provided important insights into cardiac circadian biology (32–34), these studies have exclusively focused on the LV, and the consequences of intrinsic clock dysfunction in the RV remain unexplored. Genetic or pharmacologic manipulation of clock output genes in a T1D model and in the RV would yield valuable insight into how the clock regulates RV function in health and disease.

The LV circadian program response to T1D shared features with the RV at the level of the core clock but was also distinct. In the LV, the pattern was reversed compared with the RV: *Arntl1* was unaltered across all cosinor parameters, whereas *Per2* exhibited a reduction in overall rhythmicity and a lower amplitude in T1D. This reciprocal pattern of core clock perturbation suggests that T1D does not impose a uniform core clock disruption but instead engages ventricle-specific vulnerabilities within the same feedback loop, possibly reflecting differences in posttranscriptional regulation or local metabolic signaling between the ventricles (35). Despite these differences, both ventricles converge on a common theme: the core oscillator remains largely intact while disruption is concentrated at the output layer. The core transcription-translation feedback loop (*Arntl1/Bmal1-Per/Cry*) is buffered by multiple layers of posttranslational regulation (phosphorylation, ubiquitination, and nuclear shuttling) that confer robustness against metabolic perturbation (36), whereas output genes such as *Nr1d1* and *Dbp* are more directly regulated by acute metabolic and nutrient-sensing inputs that are themselves deranged in T1D. Because *Nr1d1* and *Dbp* sit at the interface between the core clock and metabolic transcriptional programs, they may be preferentially sensitive to the metabolic milieu of T1D (hyperglycemia, insulin deficiency) even when the upstream oscillator itself continues to cycle. This possible explanation is inferential and will require direct testing in the RV and LV of T1D mice.

In the RV, *Nr1d1* and *Dbp* amplitudes were dampened, whereas in the LV, *Dbp* amplitude was elevated. This ventricle-specific inversion across the output layer has not previously been described and represents a striking novel molecular finding of this study, although the mechanistic interpretation is challenging. Elevated *Dbp* amplitude in the LV in the context of a preserved *Arntl1* waveform may reflect a compensatory response. However, whether this represents a true compensation or a maladaptive consequence of clock uncoupling warrants careful consideration. Genetic studies demonstrate that loss of *Dbp*-family output is sufficient to drive cardiac hypertrophy and ventricular dysfunction (37), but there is no evidence that a supraphysiological *Dbp* amplitude is beneficial. Moreover, dysregulation of clock output amplitude in either direction has been proposed to impair the temporal coordination of cardiac metabolic programs (38). The opposing output trajectories across the two chambers—suppression in the RV and excess in the LV—may therefore represent two distinct but equally pathological forms of circadian output dysregulation, each distorting the downstream metabolic transcriptional landscape in ventricle-specific ways that are likely to contribute independently to the functional impairments observed at the physiological level. Our data indicate that the core clock is not broken in T1D (*Arntl1* and *Per2* remain rhythmic in both chambers) but that its metabolic output arm is dysregulated. Because *Nr1d1* and *Dbp* (and the broader clock output network) coordinate the time-of-day expression of genes governing fatty acid oxidation, glucose utilization, and mitochondrial function, the dampened RV and amplified LV output rhythms would be predicted to desynchronize cardiac substrate utilization from the normal 24-h pattern of metabolic demand. Metabolic disruption could manifest as inefficient or mistimed fuel selection, for example, failure to appropriately upregulate fatty acid oxidation genes at the correct circadian phase, potentially exacerbating the metabolic inflexibility that is already a hallmark of the diabetic heart. It is worth noting that we did not directly measure cardiac substrate flux or metabolomics in this study, which is an important next step.

## LIMITATIONS AND CONCLUSIONS

Several limitations warrant consideration. This study was conducted only in male animals. However, sex is a well-established modulator of both circadian rhythms (39) and diabetic cardiomyopathy (40), thus future studies including female animals are warranted. Although the echocardiographic assessments were performed at two distinct ZT (early light and early dark phase), molecular sampling spanned the full 24-h cycle, creating an asymmetry in temporal resolution between functional and gene expression data. Additional echocardiography at more ZTs could improve our understanding of how functional rhythms vary across the day and help link changes in clock gene expression with corresponding changes in cardiac performance. All echocardiography was performed in sedated animals, and it is not clear whether autonomic and cardiac function are impacted by anesthesia in a time-of-day-dependent manner. All experiments in this study were performed in a light/dark cycle, which can mask the endogenous rhythms; thus future work will aim to perform these experiments in constant conditions to evaluate circadian rhythmicity. We acknowledge that quantification of other clock genes such as *Cry* and *Clock* would add further resolution to the core loop, but we selected a focused panel of representative genes spanning the core feedback loop (*Arntl1* as the positive-limb driver and *Per2* as a negative-limb repressor), the clock output layer (*Nr1d1, Dbp*), and clock-controlled metabolic targets (Pdk4, *Ucp3*) to characterize rhythmicity across multiple tiers of the circadian transcriptional hierarchy. Future studies warrant the analysis of other clock genes. Finally, there could be an influence of other clocks on cardiac tissue; thus, measuring rhythms in culture and measuring rhythms over a period of time is warranted. Although ex vivo culture (e.g., PER2::LUC explants) can isolate the cell-autonomous, tissue-intrinsic properties of the RV and LV clocks, this approach necessarily removes the chamber-specific hemodynamic loading, neurohumoral input, and systemic metabolic milieu present in vivo, all of which may be important drivers of the divergent RV/LV phenotypes we describe; ex vivo and in vivo approaches should therefore be viewed as complementary rather than interchangeable.

In conclusion, we show for the first time that the healthy RV exhibits robust daily rhythms in function and gene expression, revealing that the RV is under rhythmic control. We also show that T1D drives a chamber-specific rewiring of the cardiac circadian clock, rather than uniformly suppressing it. Functional analyses confirm that day-night variations in systolic and diastolic performance are impaired differently between the two ventricles. Collectively, these findings indicate that the diabetic heart does not simply lose circadian regulation but operates under a fundamentally altered temporal program in a ventricle-specific manner. We propose that chamber-specific circadian output dysregulation could contribute independently to the elevated risk of both RV and LV dysfunction reported in T1D patients, and that the timing of this dysregulation could affect the sensitivity of clinical diagnostic testing and the optimal timing of cardioprotective interventions (i.e., chronotherapy). This novel insight uncovers a previously unrecognized mechanism linking diabetes, circadian remodeling, and ventricular dysfunction, opening avenues for chronotherapy in diabetic cardiomyopathy.

## Supplementary Material

Supplemental Material

## Data Availability

All data generated during this study are included in this published article (Supplemental Material) or available upon reasonable request.
